# On Window Mean Survival Time With Interval‐Censored Data

**DOI:** 10.1002/sim.70491

**Published:** 2026-03-16

**Authors:** Takuto Iijima, Tomotaka Momozaki, Shuji Ando

**Affiliations:** ^1^ Department of Information Sciences, Graduate School of Science and Technology Tokyo University of Science Chiba Japan; ^2^ Department of Information Sciences, Faculty of Science and Technology Tokyo University of Science Chiba Japan

**Keywords:** Kaplan–Meier method, mid‐point imputation, nonparametric estimator, non‐proportional hazards, survival data

## Abstract

In recent years, cancer clinical trials have increasingly encountered non proportional hazards (NPH) scenarios, particularly with the emergence of immunotherapy. In randomized controlled trials comparing immunotherapy with conventional chemotherapy or placebo, late difference and early crossing survival curves scenarios are commonly observed. In such cases, window mean survival time (WMST), the area under the survival curve within a pre‐specified interval $\left[\tau_{0},\tau_{1}\right]$, has gained increasing attention due to its superior power compared to restricted mean survival time (RMST), the area under the survival curve up to a pre‐specified time point. Considering the increasing use of progression‐free survival as a co‐primary endpoint alongside overall survival, there is a critical need to establish a WMST estimation method for interval‐censored data; however, sufficient research has yet to be conducted. To bridge this gap, this study proposes a WMST inference method utilizing one‐point imputations and Turnbull's method. Extensive numerical simulations demonstrate that the WMST estimation method using mid‐point imputation for interval‐censored data exhibits comparable performance to that using Turnbull's method. Since the former facilitates standard error calculation, we adopt it as the standard method. Numerical simulations on two‐sample tests confirm that the proposed WMST testing method have higher power than RMST in late difference and early crossing survival curves scenarios, while having compatible power to the log‐rank test under the PH. Furthermore, even when pre‐specified $\tau_{0}$ deviated from the clinically desirable time point, WMST consistently maintains higher power than RMST in late difference and early crossing survival curves scenarios.

## 1 Introduction

In randomized controlled trials (RCTs) where the outcome is survival time, the log‐rank test has been widely used to evaluate differences in survival times between two groups. The log‐rank test is uniformly most powerful when the proportional hazards (PH) assumption holds. However, Uno et al. [1] have pointed out that the log‐rank test is closely related to the PH assumption, and when the PH assumption is clearly violated, may lack sufficient power to detect differences in survival times between two groups. Furthermore, an empirical analysis by Horiguchi et al. [2], which compare the log‐rank test with other testing methods, including restricted mean survival time (RMST) and weighted log‐rank tests, using data from recent Phase III cancer RCTs (69 trials for overall survival [OS] and 59 trials for progression‐free survival [PFS]), finds that the log‐rank test do not always exhibit superior empirical power. One potential reason for this result is the inclusion of trials in which the PH assumption does not hold. In fact, approximately 7% of the OS trials and approximately 33% of the PFS trials violated the PH assumption. In subgroup analyses focusing on trials where the PH assumption holds and PFS is the primary endpoint, the log‐rank test exhibits the highest empirical power. Additionally, a study by Trinquart et al. [3] reports that, as of 2016, 24% of oncology studies exhibited non‐proportional hazards (NPH), indicating an increasing number of cases where routine application of the log‐rank test may not be appropriate.

Based on these facts, alternative methods to the log‐rank test that do not rely on the assumption have gained renewed attention in recent years. Among them, RMST, the area under the survival curve up to a pre‐specified time point, is preferred due to its ease of clinical interpretation compared to existing statistical testing methods. Discussions on RMST have begun to appear in medical journals such as the “*Journal of Clinical Oncology*” [3], and RMST has already been used as a summary measure for primary endpoints in some studies [4, 5, 6, 7]. Although RMST is robust and interpretable even in NPH scenarios, it is not without its drawbacks. A reported limitation is its reduced power in cases where survival curves diverge in the late phase of a trial (late difference) or cross in the early phase (early crossing survival curves) [8].

A notable example of scenarios leading to late difference or early crossing survival curves is cancer immunotherapy, which has attracted significant attention in recent years. Due to its therapeutic mechanisms, many trials involving cancer immunotherapy, particularly when comparing with placebo or chemotherapy groups, have observed late differences or early crossing survival curves [9, 10]. Furthermore, the immuno‐oncology drug development pipeline has expanded rapidly, increasing approximately 2.5 times between 2017 and 2020 [11], highlighting the growing need for testing methods capable of maintaining power in late difference and early crossing survival curves scenarios. In this context, window mean survival time (WMST) [12], which is equivalent to long‐term RMST [13, 14, 15], has been proposed as a summary measure, maintaining the interpretability of RMST, outperforms RMST in cases involving late difference and early crossing survival curves. As the name suggests, WMST corresponds to the area under the survival curve within a clinically relevant window of time. Unlike RMST, WMST allows clinicians to construct a window of time during the later phase of a study when late difference or early crossing survival curves are anticipated. This enables focused analysis to determine whether the new treatment improves the mean survival time within that specific window of time. In fact, numerical simulations by Paukner and Chappell [12] demonstrate that WMST achieve higher statistical power than RMST in late difference and early crossing survival curves scenarios with right‐censored data.

In Phase III trials targeting advanced cancer, PFS, defined as the time to disease progression or death (whichever occurs first), is increasingly being used not only as a secondary endpoint but also as a primary endpoint [16, 17, 18]. For solid tumors, progression is defined by the revised RECIST guidelines [19] as tumor growth exceeding a certain threshold or the appearance of new lesions. Since progression is identified only by a medical test, such as CT, the exact time point of the progression event is unknown. Instead, it is known that the progression event occurred within the interval (l,r], where is the time of l is the last examination without progression, and r is the time when progression is confirmed. Data representing the time to an event in interval form are referred to as interval‐censored data. Kaplan–Meier (KM) method [20] cannot directly estimate survival functions using interval‐censored data. To address this, imputation methods such as right‐point imputation or mid‐point imputation are used to approximate interval‐censored observations as single time points, allowing KM methods to estimate survival functions. Additionally, Turnbull's method has been developed as a non‐parametric approach to estimate survival functions directly from interval‐censored data [21]. Although right‐point imputation is frequently used in oncology and other fields, its statistical justification remains unclear. Nishikawa and Tango [22] compare the exactness of the survival rate estimation at different times using the three methods introduced above. They find that for estimating PFS rates, the method with the smallest mean squared error (MSE) at the majority of time points is KM estimation following mid‐point imputation. However, no studies have compared these methods for estimating the accuracy of WMST.

The challenge of interval censoring extends beyond clinical trials to the rapidly expanding domain of electronic health records (EHRs) in epidemiologic research. EHRs have become invaluable sources for assembling large cohorts, with millions of patient records now being analyzed for real‐world evidence generation [23, 24]. In this setting, interval censoring is effectively inevitable since disease events can only be detected during discrete healthcare visits with irregular intervals—a fundamental characteristic that cannot be eliminated through study design. Moreover, recent EHR studies have documented non‐proportional hazards patterns, including delayed treatment effects with early crossing survival curves [25], precisely the scenarios where WMST has shown advantages over traditional methods.

Zhang et al. [8] discuss an estimator for RMST for interval‐censored data. In their approach, the survival function is estimated using Turnbull's method, and the area under the survival curve is calculated using the linear smoothing method. However, this method cannot express the standard error in a closed form, requiring the use of perturbation‐resampling methods like the bootstrap method to calculate the standard error, making the computation less straightforward. On the other hand, single‐point imputation methods, including right‐point imputation or mid‐point imputation, can express the standard error in a closed form, allowing for easier computation. In this study, we evaluate the performance of three methods for WMST estimation designed for interval‐censored data: (1) KM estimation following mid‐point imputation (mid‐point + KM), (2) KM estimation following right‐point imputation (right‐point + KM), and (3) Turnbull's estimation. Based on the results of the comparison, the most effective method is used to construct a class of WMST estimators and tests. Furthermore, this test method is compared with existing testing methods such as RMST test, the log‐rank test, and the Fleming‐Harrington test (FH test).

The remainder of this article is organized as follows. Section 2 describes the definitions of RMST and WMST, estimation methods and the testing procedure for WMST considering interval‐censored data. Section 3 conducts numerical experiments to perform one‐sample estimation and assess the exactness of the estimation. Subsequently, two‐sample tests are conducted to compare WMST with existing testing methods such as RMST, weighted log‐rank tests, and others. Additionally, under survival scenarios considering the value of $\tau_{0}$ (set prior to data collection) and the difference in the areas under two survival curves, the detection power of WMST is compared and evaluated against existing testing methods, including RMST, the log‐rank and FH tests. Section 4 applies WMST test to real‐world data and compares it with existing testing methods, as in Section 3. Finally, Section 5 provides the conclusions.

## 2 WMST for Interval‐Censored Data

This section introduces WMST, which provides interpretable results even under NPH scenarios, along with its estimation methods. RMST is presented as a special case of WMST. We describe hypothesis testing procedures using WMST and propose estimation methods specifically designed for interval‐censored data.

### 2.1 Estimation for Right‐Censored Data

Let S(t) denote the survival probability at time t≥0. WMST of survival time T is defined as the area under the survival function S(t) from $\tau_{0}$ to $\tau_{1}$ [12], that is,

$$\text{WMST}\left(\tau_{0},\tau_{1}\right)=\int_{\tau_{0}}^{\tau_{1}}S\left(t\right)\mathrm{dt}.$$

When $\tau_{0}=0$, WMST reduces to the well‐known RMST, which represents the area under the survival curve up to a specific time point $\tau_{1}$ [26], that is,

$$\text{RMST}\left(\tau_{1}\right)=\text{WMST}\left(0,\tau_{1}\right)=\int_{0}^{\tau_{1}}S\left(t\right)\mathrm{dt}.$$

Let $S_{0}\left(t\right)$ and $S_{1}\left(t\right)$ denote the survival probabilities for the control group and the treatment group, respectively. The difference in WMST between the two groups is expressed as 


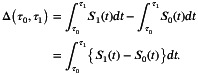




For the special case of RMST ($\tau_{0}=0$), this becomes

$$\delta \left(\tau_{1}\right)=\Delta \left(0,\tau_{1}\right)=\int_{0}^{\tau_{1}}\left\{S_{1}\left(t\right)-S_{0}\left(t\right)\right\}\mathrm{dt}.$$

With observed k ordered event times denoted as $t_{1}<\cdots <t_{k}$, the estimators of $\text{WMST}\left(\tau_{0},\tau_{1}\right)$ and $\Delta \left(\tau_{0},\tau_{1}\right)$ can be obtained as 


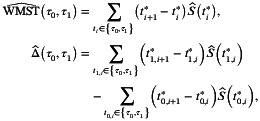


respectively, where $t_{i}^{*}=\max\left(t_{i},\tau_{0}\right)$, $t_{i+1}^{*}=\min\left(t_{i+1},\tau_{1}\right)$, $t_{j,i}^{*}=\max\left(t_{j,i},\tau_{0}\right)$, $t_{j,i+1}^{*}=\min\left(t_{j,i+1},\tau_{1}\right)$, and $t_{j,i}$ is the ordered *i*th event times in group j (j∈{0,1}). ${\hat{S}}_{0}\left(\cdot \right)$ and ${\hat{S}}_{1}\left(\cdot \right)$ denote the survival functions estimated by KM method for each group. For RMST estimation ($\tau_{0}=0$), the formulas simplify to 


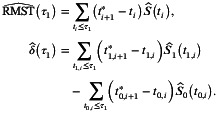

Remark 1 For selecting $\tau_{1}$, several discussions have been presented in the context of RMST and long‐term RMST [27, 28, 29, 30]. Tian et al. [30] propose a condition for empirically selecting $\tau_{1}$ as the smaller of the maximum observed times between the two groups. For selecting $\tau_{0}$, methods have been proposed to select the time point at which the two survival curves begin to diverge or the time at which WMST difference becomes significantly different from zero [31]. In this study, $\tau_{0}$ is set to a predefined value based on the anticipated survival scenario prior to the start of the trial. For example, in immunotherapy trials where delayed treatment effects are expected, $\tau_{0}$ can be informed by the anticipated onset of clinical benefit from early‐phase studies or biological considerations [12]. $\tau_{1}$ is selected as the smaller of the maximum observed times between the two groups for hypothesis testing.

### 2.2 Hypothesis Testing With WMST

The estimator for the variance of WMST can be obtained as

$$\begin{array}{ll} {\hat{\sigma }}^{2} & =\sum_{t_{i}\in \left\{\tau_{0},\tau_{1}\right\}}{\left(t_{i+1}^{*}-t_{i}^{*}\right)}^{2}\hat{\mathrm{var}}\left[\hat{S}\left(t_{i}^{*}\right)\right]\\ & \;+\sum_{t_{i}\in \left\{\tau_{0},\tau_{1}\right\}}\;\sum_{t_{j}<t_{i}}\left(t_{i+1}^{*}-t_{i}^{*}\right)\left(t_{j+1}^{*}-t_{j}^{*}\right)\hat{\mathrm{cov}}\left[\hat{S}\left(t_{i}^{*}\right),\hat{S}\left(t_{j}^{*}\right)\right], \end{array}$$

where

$$\begin{array}{cc} \hat{\mathrm{var}}\left[\hat{S}\left(t\right)\right] & \approx {\hat{S}}^{2}\left(t\right)\sum_{k|t_{k}<t}\frac{d_{k}}{r_{k}\left(r_{k}-d_{k}\right)},\\ \hat{\mathrm{cov}}\left[\hat{S}\left(t_{i}\right),\hat{S}\left(t_{j}\right)\right] & \approx \hat{S}\left(t_{i}\right)\hat{S}\left(t_{j}\right)\sum_{k|t_{k}<\min\left(t_{i},t_{j}\right)}\frac{d_{k}}{r_{k}\left(r_{k}-d_{k}\right)}, \end{array}$$

derived using Greenwood's formula, with the number of events $d_{k}$ and the number of individuals at risk $r_{k}$ (those in the risk set) at time $t=t_{k}$. Therefore, under the assumption that $S_{0}\left(t\right)$ and $S_{1}\left(t\right)$ are independent, the estimator for the variance of the difference in WMST, $\hat{\Delta }\left(\tau_{0},\tau_{1}\right)$, can be obtained as

$${\hat{\sigma }}_{\Delta }^{2}=\hat{\mathrm{var}}\left[\hat{\Delta }\left(\tau_{0},\tau_{1}\right)\right]={\hat{\sigma }}_{0}^{2}+{\hat{\sigma }}_{1}^{2},$$

where ${\hat{\sigma }}_{0}^{2}$ and ${\hat{\sigma }}_{1}^{2}$ are the estimators for the variance of WMST for each group.

When comparing WMST for the control and treatment groups, the following null hypothesis and alternative hypothesis are used:

$$H_{0}:\Delta \left(\tau_{0},\tau_{1}\right)=0,\;H_{1}:\Delta \left(\tau_{0},\tau_{1}\right)\neq 0.$$

Under the null hypothesis $H_{0}$, the following test statistic is given as

$$Z=\frac{\hat{\Delta }\left(\tau_{0},\tau_{1}\right)}{{\hat{\sigma }}_{\Delta }}.$$

This test statistic, due to the asymptotic normality of Z, can be evaluated against the standard normal distribution under large‐sample conditions.

### 2.3 WMST Estimate Designed for Interval Censoring

Since this study involves survival time data with interval censoring, KM method cannot be directly applied. To this end, we use right‐point or mid‐point imputation to handle the interval‐censored observations, estimate the survival function using KM method, and then calculate WMST. These methods are straightforward to implement in R using the existing **survWMST** package, where the wmst() function directly calculates both WMST estimates and their standard errors. Additionally, we compare these approaches with Turnbull's method [21], which directly estimates the survival function from interval‐censored data using non‐parametric maximum likelihood estimation (NPMLE) without requiring imputation, and then calculates WMST from the estimated survival function.

A key practical advantage of our proposed imputation approaches is that they enable the direct use of existing WMST software without requiring modifications to the underlying functions. In contrast, implementing WMST with Turnbull's method requires custom programming to handle the unique survival function structure produced by NPMLE, and computational techniques such as bootstrap methods are required to estimate the variance, whereas the imputation‐based methods allow for the explicit expression of standard errors. These factors make our imputation approaches considerably more practical and computationally efficient for applied researchers. Our approach to handling interval censoring is summarized as follows.

1. Mid‐point + KM: The survival function is estimated with KM method after imputing the interval‐censored observations $\left(l_{i},r_{i}\right]$ by
$$t_{i}=\frac{l_{i}+r_{i}}{2}.$$

2 Right‐point + KM: The survival function is estimated with KM method after imputing the interval‐censored observations $\left(l_{i},r_{i}\right]$ by
$$t_{i}=r_{i}.$$

3 Turnbull's algorithm: Let $t_{i}$ (i=1,…,n) denote survival times that follow the survival function S(⋅), where the interval‐censored observation of $t_{i}$ is given as the interval $\left(l_{i},r_{i}\right]$. Then the likelihood function can be expressed as
$$L\left(S\right)\propto \prod_{i=1}^{n}\mathrm{Pr}(t_{i}\in \left(l_{i},r_{i}]\right)=\prod_{i=1}^{n}\left\{S\left(l_{i}\right)-S\left(r_{i}\right)\right\}$$
  and the survival function is estimated by maximizing this likelihood. For computational implementation, we use the icfit() function from the R package interval [32], which computes the NPMLE via Turnbull's iterative algorithm.

Compared to the Turnbull method, the methods combining mid‐point and right‐point imputations with KM estimation are easier to implement, requiring only simple data preprocessing.

## 3 Simulation Studies

This section begins with a description of the settings common to all numerical simulations. It then outlines the settings and results used to evaluate estimation accuracy, as well as those for assessing test size and power. Finally, to examine the robustness of WMST with interval‐censored data, the section investigates the impact of RMST difference δ, the starting point of WMST, $\tau_{0}$, and the divergence or crossing point of the survival curves x on the tests, focusing on survival scenarios frequently observed in cancer immunotherapy trials, such as early crossing survival curves and late difference.

### 3.1 Simulation Settings

The interval‐censored data used in the simulations are generated following Zhang et al. [8]. The procedure for simulating a scenario where interval censoring arises in PFS is described below. For each subject, a baseline examination is conducted at time $g_{0}$, followed by K periodic follow‐up examinations $\left\{g_{k}=g_{0}+k\times 1/\left(K+1\right)\right\}$ at equally spaced intervals. Additionally, to account for the practical situation in clinical trials where subjects may miss scheduled visits, a dropout probability vector $P_{\text{dropout}}$ of length K is introduced. It is assumed that all subjects complete the baseline examination. Furthermore, to investigate how the proportion of interval censoring in observed events impacts the performance of WMST, a parameter $p_{\text{exact}}$ is introduced. The following steps are repeated for i=1,…,n.

Step 1. Generate the baseline examination time $g_{0\left(i\right)}$ from Unif(0,1/(K+1)) and construct the examination schedule $\left\{g_{0\left(i\right)},g_{1\left(i\right)},\ldots ,g_{K\left(i\right)}\right\}$.

Step 2. Generate the event time $t_{i}$ from a specific distribution described later. Additionally, based on the dropout probability vector $P_{\text{dropout}}$, determine whether the examination is missed at time $g_{j\left(i\right)}$ (j=1,…,K). Generate a random flag $\xi_{i}$ from $\text{Bernoulli}\left(p_{\text{exact}}\right)$ to specify whether the event is death ($\xi_{i}=1$) or progression ($\xi_{i}=0$).

Step 3. Based on the random flag $\xi_{i}$, determine the type of event and construct the interval‐censored observation:

- If $\xi_{i}=1$ (death event): Set the observed interval as $\left(l_{i},r_{i}\right]=\left(t_{i}-\epsilon ,t_{i}\right]$, where ϵ is a small positive number close to 0. This represents an exact event time.
- If $\xi_{i}=0$ (progression event): Identify the examination interval containing the true event time $t_{i}$. Specifically, find consecutive examination times $g_{k\left(i\right)}$ and $g_{l\left(i\right)}$ (where k<l) such that $g_{k\left(i\right)}<t_{i}\leq g_{l\left(i\right)}$ and neither examination was missed due to dropout. Set the observed interval as $\left(l_{i},r_{i}\right]=\left(g_{k\left(i\right)},g_{l\left(i\right)}\right]$. This simulates the clinical scenario where progression is detected at examination $g_{l\left(i\right)}$ but was not present at the previous examination $g_{k\left(i\right)}$.

Step 4. If the event occurs after the final examination time $g_{K\left(i\right)}$ or if the progression event cannot be confirmed due to missed examinations, set $r_{i}=\infty$ and treat it as right‐censored.

### 3.2 Evaluation of Estimation

In the numerical simulations for evaluating estimation methods, various parameter settings are examined to assess the performance of WMST estimator, taking interval censoring into account. First, for the dropout probability vector $P_{\text{dropout}}$, the following four dropout scenarios are considered. In this case, the probability of missing any given examination, except for the final examination, is assumed to be equal across all follow‐up examinations, while the dropout probability for the final examination is set to be twice as high. Namely,

- *None* $:P_{\text{dropout},k}=0$ for k=1,…,K;
- *Low* $:P_{\text{dropout},k}=0.1$ for k=1,…,K−1 and $P_{\text{dropout},K}=0.2$;
- *Medium* $:P_{\text{dropout},k}=0.2$ for k=1,…,K−1 and $P_{\text{dropout},K}=0.4$;
- *High* $:P_{\text{dropout},k}=0.3$ for k=1,…,K−1 and $P_{\text{dropout},K}=0.6$.

Event times have the Weibull distribution with the probability density function

$$f_{\text{Weibull}}\left(t\right)=\frac{{\mathrm{pt}}^{p-1}}{\lambda^{p}}\exp\left(-{\frac{t}{\lambda }}^{p}\right)\;\left(\lambda >0\right).$$

**FIGURE 1:**
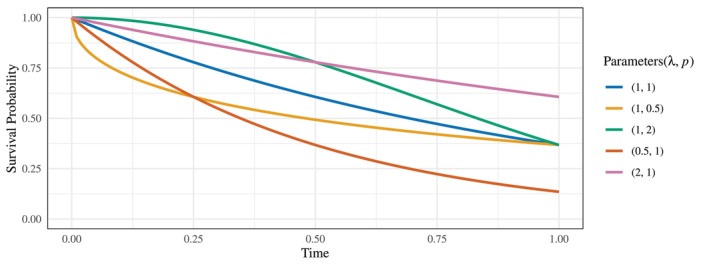
Survival curves of five Weibull distributions with parameters (λ,p)∈{(1,1),(1, 0.5),(1,2),(0.5, 1),(2,1)}. These true distributions, differentiated by color, represent constant, decreasing, and increasing hazard scenarios used for generating simulated data in the estimation evaluation.

We considered the five combinations of scale parameter λ and shape parameter p

(λ,p)∈{(1,1),(1,0.5),(1,2),(0.5,1),(2,1)},

taking into account survival scenarios where the risk of death is constant, decreasing, or increasing over time, as well as scenarios with high or low mortality risk. Figure 1 shows the survival curves of the Weibull distributions examined.

The simulation parameters also include sample sizes n∈{100, 200, 400}, the number of follow‐up examinations K∈{3, 5, 10, 20}, and the proportion of death events among all observed events $p_{\text{exact}}\in \left\{0, 0.2, 0.5, 1.0\right\}$. The numerical simulations are conducted with the event time distribution set to Weibull(λ=1,p=1), the dropout probability vector $P_{\text{dropout},k}$ set to *Medium*, n=100, K=5, and $p_{\text{exact}}=0$ as a default setting. The influence of each parameter is evaluated by varying one parameter at a time while keeping the others fixed. For the WMST estimation evaluation, we examine two different values of $\tau_{0}$ (0.25 and 0.50), and $\tau_{1}$ is selected as the smaller of the maximum observed times between the two groups [30]. For each parameter configuration, 10 000 numerical simulations are performed, and the estimation accuracy is examined in terms of relative bias (rBias) and mean squared error (MSE)

$$\begin{array}{ll} & \text{rBias}=\frac{\sum_{s=1}^{10000}{\hat{\mu }}_{s}\left(\tau_{0},\tau_{1}\right)-\mu \left(\tau_{0},\tau_{1}\right)}{\mu \left(\tau_{0},\tau_{1}\right)},\\ & \mathrm{MSE}=\frac{1}{10000}\sum_{s=1}^{10000}{\left({\hat{\mu }}_{s}\left(\tau_{0},\tau_{1}\right)-\mu (\tau_{0},\tau_{1})\right)}^{2}, \end{array}$$

where ${\hat{\mu }}_{s}\left(\tau_{0},\tau_{1}\right)$ is estimated WMST during the *s*th numerical simulation.

Table 1 shows that the mid‐point + KM and Turnbull had a smaller MSE compared to right‐point + KM overall. However, the difference in MSE between mid‐point + KM and Turnbull is negligible. When $\tau_{0}=0.50$, the difference in MSE between the mid‐point + KM and Turnbull is also negligible, but in terms of the absolute value of rBias, Turnbull outperforms the mid‐point + KM. Regarding the distribution of survival time T, for Weibull(λ=1,p=2), MSE is nearly identical between the mid‐point + KM and Turnbull, but Turnbull shows higher accuracy than the mid‐point + KM in terms of the absolute value of rBias. Conversely, for Weibull(λ=0.5,p=1), mid‐point + KM outperforms Turnbull in the absolute value of rBias.

**TABLE 1:** Simulation results for the estimation of interval‐censored WMST with $\tau_{0}\in \left\{0.25, 0.50\right\}$ using three methods: mid‐point imputation + KM, right‐point imputation + KM, and Turnbull method.

	$\tau_{0}=0.25$						$\tau_{0}=0.50$					
	Mid‐point + KM		Right‐point + KM		Turnbull		Mid‐point + KM		Right‐point + KM		Turnbull	
Parameter	rBias	MSE	rBias	MSE	rBias	MSE	rBias	MSE	rBias	MSE	rBias	MSE
T∼ Weibull (λ, p)												
Weibull (1, 1)^a^	−0.0068	**0.0011**	0.0949	0.0024	−0.0065	**0.0011**	−0.0147	**0.0006**	0.0727	0.0008	−0.0104	**0.0006**
Weibull (1, 0.5)	−0.0061	**0.0012**	0.0727	0.0018	−0.0084	0.0013	−0.0149	**0.0006**	0.0382	**0.0006**	−0.0122	**0.0006**
Weibull (1, 2)	−0.0111	0.0008	0.0848	0.0024	−0.0038	**0.0007**	−0.0168	**0.0005**	0.0965	0.0012	−0.0079	**0.0005**
Weibull (0.5, 1)	−0.0047	**0.0008**	0.2397	0.0039	−0.0116	**0.0008**	−0.0243	**0.0004**	0.2032	0.0009	−0.0190	**0.0004**
Weibull (1, 2)	−0.0057	**0.0008**	0.0393	0.0040	−0.0046	0.0009	−0.0100	**0.0005**	0.0266	**0.0005**	−0.0075	**0.0005**
$P_{\text{dropout}}$												
None	−0.0041	**0.0010**	0.0739	0.0019	−0.0053	0.0011	−0.0074	**0.0005**	0.0642	0.0008	−0.0079	**0.0005**
Low	−0.0052	**0.0010**	0.0862	0.0022	−0.0055	0.0011	−0.0102	**0.0006**	0.0717	0.0008	−0.0084	**0.0006**
High	−0.0095	**0.0011**	0.0957	0.0024	−0.0093	0.0012	−0.0211	**0.0006**	0.0605	0.0007	−0.0151	**0.0006**
n												
200	−0.0026	**0.0005**	0.0969	0.0020	−0.0032	**0.0005**	−0.0078	**0.0003**	0.0759	0.0006	−0.0051	**0.0003**
400	−0.0006	**0.0003**	0.0977	0.0018	−0.0017	**0.0003**	−0.0046	**0.0001**	0.0771	0.0005	−0.0027	**0.0001**
K												
3	−0.0092	**0.0011**	0.1111	0.0029	−0.0097	0.0012	−0.0239	0.0007	0.0762	0.0009	−0.0150	**0.0006**
10	−0.0028	**0.0010**	0.0602	0.0015	−0.0026	**0.0010**	−0.0057	**0.0005**	0.0516	0.0006	−0.0044	**0.0005**
20	−0.0015	**0.0010**	0.0329	0.0012	−0.0014	**0.0010**	−0.0032	**0.0005**	0.0297	0.0006	−0.0027	**0.0005**
$P_{\text{exact}}$												
0.2	−0.0132	**0.0011**	0.0691	0.0017	−0.0118	**0.0011**	−0.0249	**0.0006**	0.0466	**0.0006**	−0.0198	**0.0006**
0.5	−0.0219	**0.0011**	0.0303	**0.0011**	−0.0205	**0.0011**	−0.0386	0.0006	0.0074	**0.0005**	−0.0346	0.0006
1.0	−0.0339	**0.0012**	−0.0339	**0.0012**	−0.0339	**0.0012**	−0.0576	**0.0007**	−0.0576	**0.0007**	−0.0576	**0.0007**

*Note:* Bold values indicate the smallest mean squared error for each scenario.

Abbreviations: MSE, mean squared error; rBias, relative bias.

^a^ The default simulation setting is the n=100, K=5, $p_{\text{exact}}=0$, T∼Weibull(1,1) and *Medium* dropout rate.

This pattern can be explained by differences in the survival time distributions. Among the five distributions examined, Weibull(λ=1,p=2) is characterized by increasing hazards toward the later stages of the trial. Under such conditions, Turnbull demonstrates higher accuracy in terms of the absolute value of rBias. To further compare the estimation accuracy of the mid‐point + KM and Turnbull under a distribution with even higher hazards in the later stages, Weibull(λ=1,p=3) is also considered. The results show that while the MSE values are almost identical, Turnbull demonstrates higher accuracy in terms of the absolute value of rBias compared to the mid‐point + KM. In the most notable case among the 10 000 numerical simulations, Turnbull accurately estimates the true survival curve, whereas the mid‐point + KM underestimates the survival curve for most of the trial duration, particularly near the end of the trial period, leading to a significant divergence. This discrepancy likely contributes to the increase in the absolute value of rBias for the mid‐point + KM. However, in the field of cancer immunotherapy, where WMST is particularly relevant, survival scenarios such as Weibull(λ=1,p=2), with increasing hazards in the last part of the trial period, are considered uncommon.

As n increases, the absolute value of rBias decreases for both the mid‐point + KM and Turnbull, while it shows little change for the right‐point + KM. When the parameter K decreases, the interval width of the interval‐censored data increases. However, even with small values of K, the MSE values for the mid‐point + KM and Turnbull remains nearly unchanged. Similarly, the parameter $P_{\text{dropout}}$, which also affects the width of the interval, shows little impact on the MSE values for the mid‐point + KM and Turnbull. In contrast, for the right‐point + KM, the MSE values varies depending on changes in K and $P_{\text{dropout}}$. Furthermore, even with an increased proportion of interval censoring, the MSE values for the mid‐point + KM and Turnbull remains nearly constant.

Based on these simulation results, we recommend the mid‐point + KM method for practical applications. Despite achieving comparable estimation accuracy to Turnbull's method, the mid‐point + KM approach offers substantial practical advantages. As detailed in Section 2.3, implementation requires only simple interval midpoint calculation followed by direct application of the well‐established wmst() function in the **survWMST** package. In contrast, implementing WMST with Turnbull's method requires researchers to compute the NPMLE using appropriate software and then develop custom code to process the resulting survival function structure, which is a non‐trivial task for applied researchers.

Furthermore, the mid‐point + KM method provides closed‐form variance estimates. Turnbull's method, conversely, requires bootstrap resampling for variance estimation to obtain accurate variance estimates, substantially increasing computational burden. Conducting the hypothesis testing evaluations in Sections 3.3 and 3.4 with bootstrap‐based variance estimation would have required prohibitive computational time. Additionally, implementing and interpreting bootstrap procedures correctly requires statistical expertise that may present barriers for some applied researchers. Therefore, the mid‐point + KM method offers the optimal balance of statistical rigor and practical utility, making it our recommended approach for WMST estimation with interval‐censored data.

### 3.3 Evaluation of Hypothesis Test

The mid‐point imputation method is used throughout the following hypothesis testing evaluations, as it provides the best balance between statistical accuracy (Section 3.2) and computational efficiency (Section 2.3) required for our comprehensive simulation studies. The survival function estimated using KM method is then used to calculate WMST, and hypothesis testing based on WMST is evaluated.

Harrington and Fleming [33] introduce FH test as a type of weighted log‐rank test (WRT). FH test uses the weight function

$$w_{t}\left(p,q\right)=\hat{S}{\left(t-\right)}^{p}{\left(1-\hat{S}\left(t-\right)\right)}^{q},$$

for p≥0 and q≥0, where $\hat{S}\left(t-\right)$ represents the left‐continuous survival function estimated at time t using the pooled survival time data from both groups using KM method. By appropriately selecting the hyperparameters p and q, it is possible to assign greater weight to differences occurring in either the early or late stages of the study period. In this study, we focus on survival scenarios such as late difference and early crossing survival curves, where the survival curves of the two groups diverge significantly in the later stages of the trial. Therefore, the hyperparameters are set to p=0 and q=1.

In addition to the single weighted log‐rank tests, combination tests have gained attention as robust alternatives under non‐proportional hazards. The MaxCombo test and related work [34, 35, 36, 37] combines multiple FH weighted log‐rank statistics to achieve robust power across various survival scenarios. Specifically, MaxCombo typically uses the maximum of test statistics from FH (0,0), FH (0,1), FH (1,0), and FH (1,1), which are sensitive to proportional hazards, late differences, early differences, and middle differences, respectively [38]. This versatile testing approach has become increasingly common in immunotherapy trials where the timing of treatment benefit is uncertain [39].

Under the null and alternative hypotheses, 10 000 numerical simulations are repeated to calculate and evaluate the test size or power. Following the settings of Pan [40], the numerical simulation of the two‐sample WMST test is conducted under the PH and NPH assumptions. Similarly to the evaluation of the estimation methods, the generation of interval‐censored data follows the method of Zhang et al. [8]. However, in this subsection, all simulation settings are based on the default settings from the previous section, with only the distribution parameters for survival time T being replaced.

**FIGURE 2:**
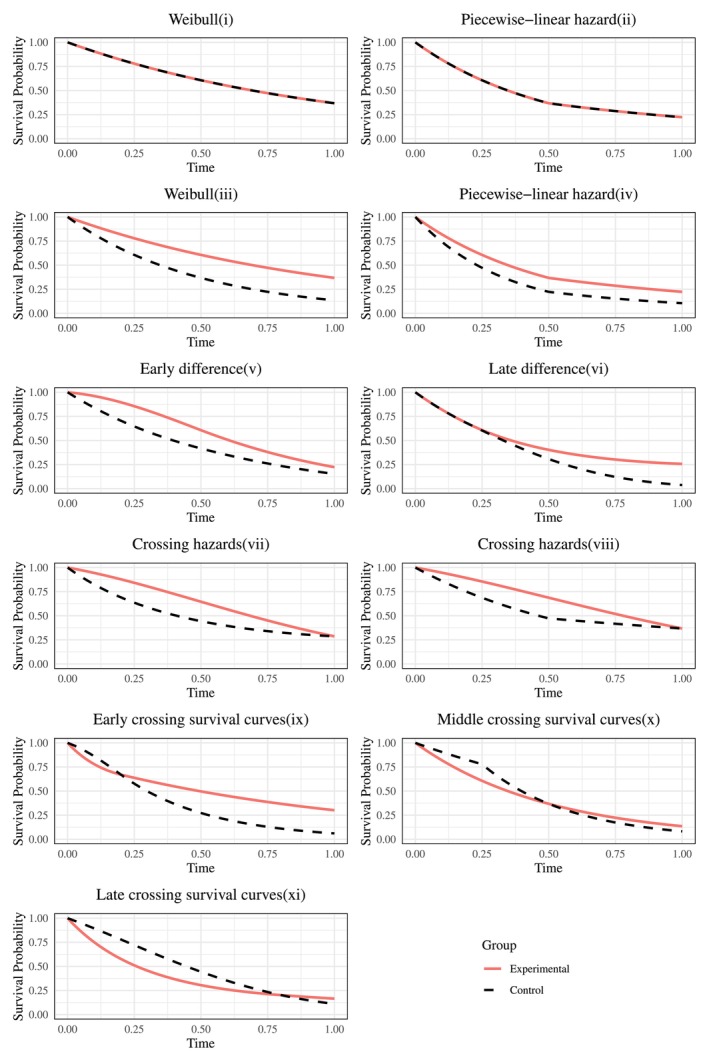
The survival time scenarios used in the numerical simulations for hypothesis testing. $H_{0}$: (i)–(ii), $H_{1}\left(\mathrm{PH}\right)$: (iii)–(iv), $H_{1}\left(\mathrm{NPH}\right)$: (v)–(xi).

Figure 2 illustrates the survival scenarios under the null and alternative hypotheses and demonstrates the diverse patterns of treatment effects that can occur in clinical practice. The null hypothesis scenarios show identical survival curves between control and experimental groups: (i) exponential distribution and (ii) piecewise‐linear hazard, serving as baselines for evaluating Type I error control. Additional null scenarios—including decreasing (p=0.5) and increasing (p=2) Weibull hazards, and time‐dependent piecewise‐linear hazards—are presented in Appendix A for comprehensive validation across diverse baseline conditions. The alternative hypotheses encompass both proportional hazards (PH) and non‐proportional hazards (NPH) patterns. The PH scenarios (iii–iv) include Weibull distributions with constant hazard ratio $\lambda_{1}^{p}/\lambda_{0}$ and piecewise‐linear hazard functions maintaining a hazard ratio of 1.5 throughout the study period. Additional PH scenarios with varying shape parameters and time‐dependent hazards are detailed in Appendix A.

Most importantly for our study, the NPH scenarios (v–xi) are classified as early difference, late difference, crossing hazards, and crossing survival curves, capturing the complex survival patterns frequently observed in cancer immunotherapy trials [41, 42, 43, 44, 45]. The early difference scenario (v) shows initial treatment benefit that diminishes over time, while the late difference scenario (vi) represents delayed treatment effects that become apparent only after an initial period—a hallmark of immunotherapy responses. The crossing hazards scenarios (vii–viii) exhibit hazard functions that intersect over time, leading to changing hazard ratios throughout the study period. The crossing survival curves scenarios (ix–xi) are particularly relevant to immunotherapy studies, where initial harm or lack of benefit may be followed by substantial long‐term advantages. These diverse scenarios enable comprehensive evaluation of WMST's performance across clinically relevant survival patterns, particularly those challenging for traditional methods. The distributions or hazard functions of survival times under each scenario for both the null and alternative hypotheses are as follows.

- Null hypotheses
  – PH
    ∘ Weibull
      i. $T_{0},T_{1}∼\text{Weibull}\left(\lambda =1,p=1\right)$
    ∘ Piecewise‐linear hazard
      ii. $h_{0}\left(t\right)=h_{1}\left(t\right)=2$ (t∈[0,0.5)); $h_{0}\left(t\right)=h_{1}\left(t\right)=1$ (t∈[0.5,1])
- Alternative hypotheses
  – PH
    ∘ Weibull
      iii. $T_{0}∼\text{Weibull}\left(\lambda =0.5,p=1\right),\;T_{1}∼\text{Weibull}\left(\lambda =1,p=1\right)$
    ∘ Piecewise‐linear hazard
      iv. $h_{0}\left(t\right)=3,\;h_{1}\left(t\right)=2$ (t∈[0,0.5)); $h_{0}\left(t\right)=1.5,h_{1}\left(t\right)=1$ (t∈[0.5,1])
  – NPH
    ∘ Early difference
      v. $h_{0}\left(t\right)=1.75$, $h_{1}\left(t\right)=3t+0.25$ (t∈[0,0.5)); $h_{0}\left(t\right)=h_{1}\left(t\right)=t+1.25$ (t∈[0.5,1])
    ∘ Late difference
      vi. $h_{0}\left(t\right)=h_{1}\left(t\right)=2$ (t∈[0,0.2)); $h_{0}\left(t\right)=4t+1.2$, $h_{1}\left(t\right)=-2t+2.4$ (t∈[0.2,1])
    ∘ Crossing hazards
      vii. $h_{0}\left(t\right)=-1.5t+2$, $h_{1}\left(t\right)=1.5t+0.5$ (t∈[0,1])
      viii. $h_{0}\left(t\right)=1.5$, $h_{1}\left(t\right)=t+0.5$ (t∈[0,0.5)); $h_{0}\left(t\right)=0.5$, $h_{1}\left(t\right)=t+0.5$ (t∈[0.5,1])
    ∘ Crossing survival curves
      ix. Early: $h_{0}\left(t\right)=10t+1$, $h_{1}\left(t\right)=-10t+3$ (t∈[0,0.2)); $h_{0}\left(t\right)=3$, $h_{1}\left(t\right)=1$ (t∈[0.2,1])
      x. Middle: $h_{0}\left(t\right)=1$, $h_{1}\left(t\right)=2$ (t∈[0,0.25)); $h_{0}\left(t\right)=3$, $h_{1}\left(t\right)=2$ (t∈[0.25,1])
      xi. Late: $h_{0}\left(t\right)=2.5t+1$, $h_{1}\left(t\right)=-2.5t+3$, (t∈[0,0.8)); $h_{0}\left(t\right)=3$, $h_{1}\left(t\right)=1$ (t∈[0.8,1])

**FIGURE 3:**
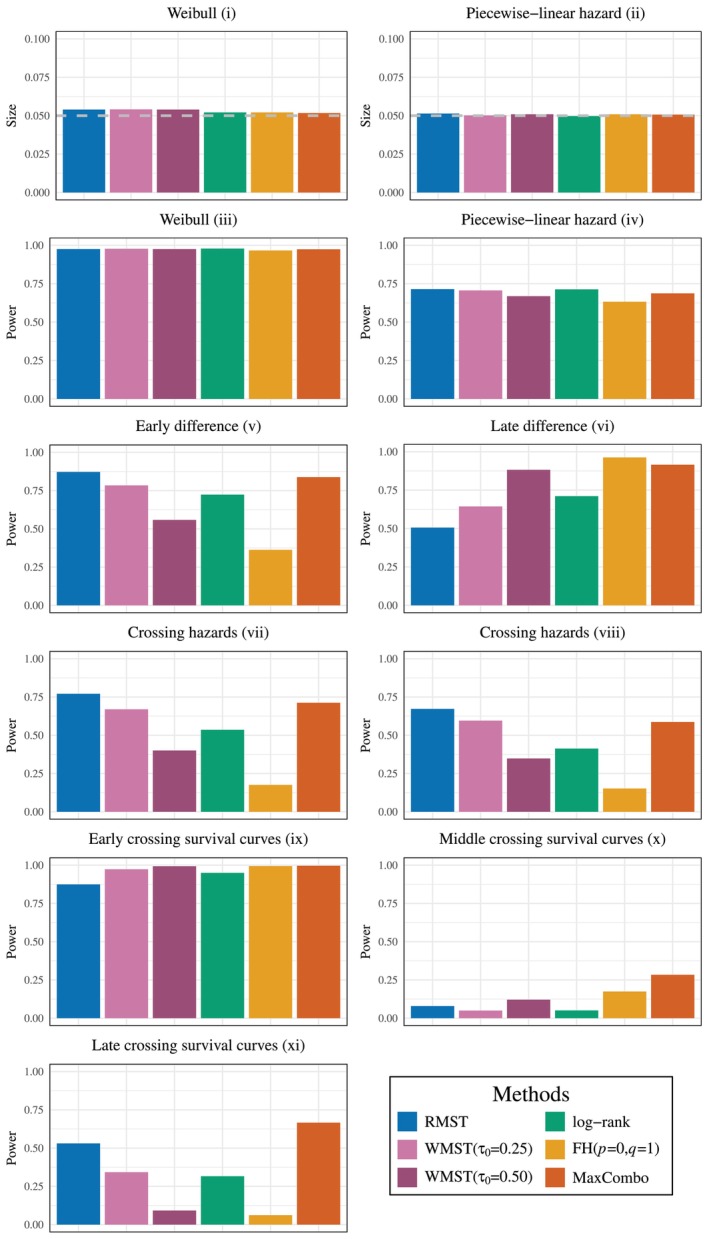
Size and power comparison of WMST tests with existing methods (RMST, Log‐rank, FH, and MaxCombo) across 11 survival scenarios. $H_{0}$: (i), (ii), $H_{1}$ (PH): (iii), (iv), $H_{1}$ (NPH): (v)–(xi) [complete results in Appendix B].

Figure 3 presents a comprehensive comparison of test performance across various survival scenarios. Under the null hypothesis scenarios (i) and (ii), all testing methods maintain Type I error rates close to the nominal significance level of α=0.05.

Under the alternative hypothesis with proportional hazards (PH) assumptions in scenarios (iii) and (iv), as well as in all PH scenarios presented in Appendix A, the log‐rank test exhibits the highest statistical power, as expected given its optimality under PH. Notably, both WMST and RMST tests achieve power levels remarkably close to the log‐rank test, while consistently outperforming FH (p=0,q=1) and MaxCombo tests across all PH scenarios. This consistent superiority of WMST over FH and MaxCombo under PH represents a crucial advantage, as PH violations in practice are often less extreme than anticipated during trial design. The slightly reduced power of MaxCombo relative to the optimal test (which in PH scenarios is the log‐rank test, equivalent to FH (0,0)) is consistent with the findings of Lin et al. [37], attributable to the inherent model selection penalty when combining multiple test statistics.

In non‐proportional hazards (NPH) scenarios, MaxCombo demonstrates superior power in most cases, which is expected given its design to combine multiple weight functions (FH (0,0), FH (0,1), FH (1,0), and FH (1,1)) that are sensitive to different patterns of survival differences [38]. For late difference scenarios such as (vi), where treatment effects manifest predominantly in later follow‐up periods, the FH (0,1) test, which assigns greater weight to later events, achieves the highest power. Similar to the PH scenarios, MaxCombo shows slightly reduced power compared to the optimal single test due to the multiplicity adjustment inherent in combination testing.

Of particular interest are the late difference (vi) and early crossing survival curves (ix) scenarios, where WMST consistently outperforms RMST. Furthermore, with appropriate selection of $\tau_{0}$, WMST achieves power comparable to both FH (0,1) and MaxCombo tests while maintaining its interpretability advantage. This demonstrates that WMST can effectively balance statistical efficiency with clinical interpretability in scenarios where treatment effects are delayed or when survival curves cross.

In contrast, for scenarios including early difference (v), crossing hazards (vii, viii), and late crossing survival curves (xi), we observe a consistent power hierarchy: RMST > WMST ($\tau_{0}=0.25$) > WMST ($\tau_{0}=0.50$). This pattern can be attributed to the decreasing magnitude of the WMST difference between treatment groups as $\tau_{0}$ increases (see Table B1 in Appendix B), making treatment effects more difficult to detect. Similarly, in the middle crossing survival curves scenario (x), the minimal WMST difference of 0.003 results in correspondingly low statistical power, highlighting the importance of window selection in relation to the anticipated timing of treatment effects. Notably, even in these scenarios where WMST underperforms relative to RMST, FH (0,1) tests exhibit even lower power. Beyond these power limitations, FH tests suffer from additional critical drawbacks: the lack of interpretable effect measures [37, 39] and elevated false positive rates in crossing hazards scenarios [12, 46]. These comprehensive limitations demonstrate that WMST maintains both statistical and methodological advantages over weighted log‐rank approaches.

These power comparisons reveal an important trade‐off in test selection. While MaxCombo and optimally‐selected FH tests may achieve higher power in certain NPH scenarios, they provide only p‐values and test statistics without clinically meaningful effect estimates [37, 39, 47]. In contrast, WMST provides the mean survival time within a specified window, which is easier to interpret compared to the hazard ratio under the NPH [48, 49, 50, 51]. This enables clinicians to communicate treatment effects clearly—for example, “treatment A provides an average of X additional months of survival between months Y and Z.” This advantage extends beyond clinical trials and enables even clinicians or patients without advanced statistical knowledge to make informed and confident decisions about treatment plans or drug choices based on a clear understanding.

Given that test selection must be pre‐specified before trial commencement, WMST represents a practical choice that balances statistical performance with clinical utility. It maintains competitive power across diverse survival patterns—consistently outperforming weighted tests under PH and achieving reasonable power under various NPH scenarios, while providing interpretable effect estimates essential for regulatory decisions and clinical practice. This combination of stable statistical performance and direct clinical interpretability positions WMST as particularly valuable in modern clinical trials where clear communication of treatment benefits is as important as their detection.

### 3.4 Altering Choice of $\tau_{0}$, Crossing Point and Difference of RMST

The setting of $\tau_{0}$ is determined prior to the collection of trial data in actual clinical trials. In this study, numerical simulations are conducted to investigate how the pre‐specified value of $\tau_{0}$ affects the power to detect differences when the divergence or crossing point of the two survival curves derived from the trial data occurs at different points. In addition, the impact of the true difference in RMST on power is examined. Focusing on the late difference and early crossing survival curves scenarios, where WMST shows superior results to RMST in the previous subsection, the divergence or crossing point x of the two survival curves is set at {0.10,0.15,0.20,0.25,0.30}, $\tau_{0}$ at {0.05,0.10,0.15,0.20,0.25,0.30,0.35,0.40,0.45,0.50}, and the difference of RMST δ is set at {0.10,0.15,0.20}. For all 5×10×3 combinations, 5000 numerical simulations are conducted to calculate the power. As in the previous subsection, all other parameters are kept in their default settings.

**FIGURE 4:**
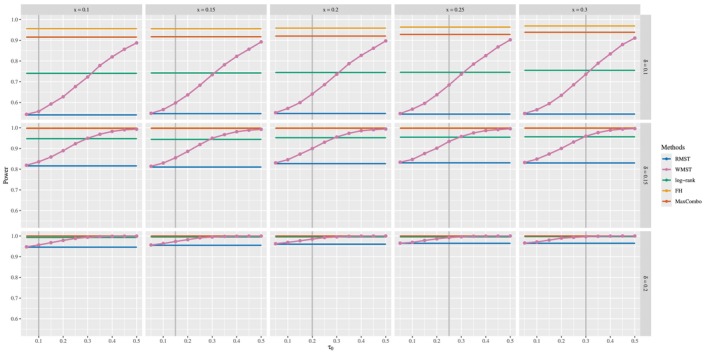
Power for WMST tests based on $\tau_{0}$, the divergence point (x) of survival curves and difference of RMST under the late difference scenario, compared to three existing methods: RMST, log‐rank, FH, and MaxCombo [complete results in Appendix B].

**FIGURE 5:**
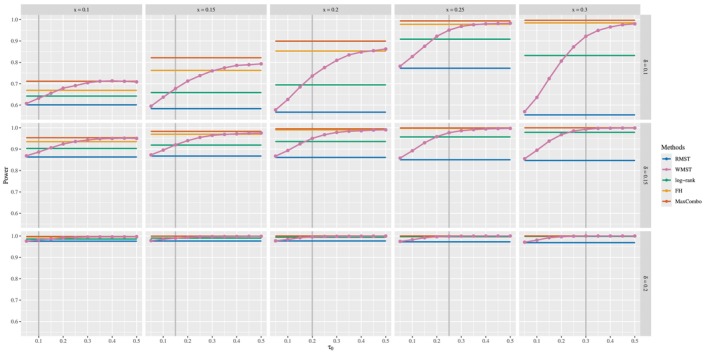
Power for WMST tests based on $\tau_{0}$, the crossing point (x) of survival curves and difference of RMST under the early crossing survival curves scenario, compared to three existing methods: RMST, log‐rank, FH, and MaxCombo [complete results in Appendix B].

Figures 4 and 5 present comprehensive power comparisons for the late difference and early crossing survival curves scenarios, respectively, revealing distinct patterns that inform optimal window selection strategies for WMST analysis. In Figure 4 (late difference scenario), several critical observations emerge. First, WMST (pink line) consistently demonstrates superior power compared to RMST (blue line) across all combinations of parameters, with the WMST power curves consistently positioned above those for RMST regardless of the values of $\tau_{0}$ and x. While this superiority is maintained throughout, the absolute power of WMST increases as $\tau_{0}$ increases, reflecting the benefit of focusing the analysis window on later time periods where treatment differences are more pronounced in the late difference scenario. Second, the magnitude of the difference of RMST δ dramatically influences the power of both methods, with larger values of δ leading to substantial power gains. The vertical progression across panels in Figure 4 shows that as δ increases from 0.10 to 0.20, the power curves shift upward dramatically, with WMST achieving near‐perfect power (>0.99) for δ=0.20 when $\tau_{0}$ is sufficiently large (typically $\tau_{0}\geq 0.30$), regardless of the divergence point x. Notably, while RMST (blue line) also improves with increasing δ, its power remains consistently lower than WMST, and this gap persists across all effect sizes. Third, a notable feature evident in Figure 4 is the robustness of WMST to variations in the divergence point x. Examining horizontally across the panels for fixed δ, the WMST power curves (pink) maintain similar shapes and levels despite changes in x. This property has important practical implications: in actual clinical trials, the precise timing when treatment effects begin to manifest (x) is unknown at the design stage. The robustness of WMST to variations in x provides protection against this uncertainty, maintaining statistical power regardless of when the survival curves actually diverge.

Figure 5 (early crossing survival curves scenario) reveals distinctly different power dynamics. When δ=0.10, WMST achieves moderate power levels that depend both on $\tau_{0}$ and the crossing point x. In contrast, as the effect size increases (δ=0.15 and δ=0.20), WMST (pink) achieves consistently high power (often exceeding 0.90) with a much weaker dependence on the specific values of $\tau_{0}$ and x, providing substantial tolerance for uncertainty in window placement. Importantly, when the effect size is small (δ=0.10), setting $\tau_{0}$ before the crossing point x can substantially reduce the power, potentially leading to false negative results—failing to detect a treatment effect that actually exists. This reduction occurs because the analysis window primarily captures the pre‐crossing period where the treatment group performs worse, diluting or masking the subsequent modest benefit. Therefore, when early crossing is anticipated with small‐to‐moderate treatment effects, choosing a conservative (later) value for $\tau_{0}$ may be preferable to avoid missing true treatment effects. Throughout all effect sizes and crossing points, WMST maintains its consistent advantage over RMST (blue), with the gap between them generally widening as $\tau_{0}$ increases.

Examining the performance patterns across Figures 4 and 5 reveals important insights about the relative strengths of different testing methods. When $\tau_{0}\geq x$, WMST (pink) generally outperforms the log‐rank test (green) in both late difference and early crossing scenarios, demonstrating the advantage of focusing the analysis window on periods where treatment effects are most pronounced. The FH (0,1) (yellow) and MaxCombo (orange) tests represent the current best practices for handling non‐proportional hazards, achieving consistently high power across both scenarios. Remarkably, when $\tau_{0}\geq x$, WMST achieves power comparable to these optimized methods and can even exceed FH performance, especially in the early crossing scenario. This competitive statistical power becomes more consistent as the effect size increases, with WMST approaching MaxCombo's performance for δ=0.20. Notably, RMST (blue) fails to achieve this level of performance, highlighting the substantial efficiency gain from focusing on a carefully selected time window. Crucially, WMST achieves this strong statistical performance while providing a directly interpretable expected survival time within the window, which rank‐based methods cannot offer. This combination of competitive power and clinical interpretability positions WMST as an attractive method for analyzing interval‐censored data under non‐proportional hazards, offering both the rigor needed for regulatory decisions and the clarity needed for clinical communication.

## 4 Application

**FIGURE 6:**
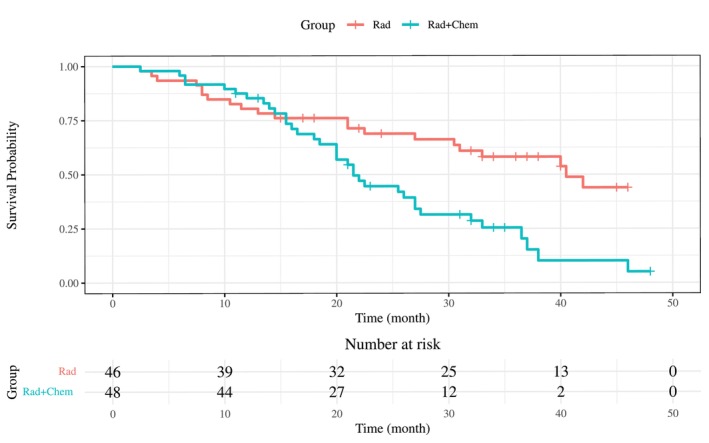
KM curves estimated by using the mid‐point imputation method on interval‐censored data in the BCOS dataset, where “Rad” represents the radiotherapy group and “Rad + Chem” represents the group that received radiotherapy with adjuvant chemotherapy.

This section demonstrates the results of applying the WMST method to real data with interval censoring. Similarly to the previous section, the interval‐censored data are imputed using the mid‐point imputation method, after which the survival function is estimated with KM method. WMST is then estimated and tested, and the results are compared with existing testing methods. The real data used in this study are from the “bcos” dataset available in the interval package of R software [52]. The bcos dataset contains interval‐censored survival times for 46 patients who underwent radiation therapy and 48 patients who received a combination of radiation therapy and chemotherapy, with the survival endpoint defined as cosmetic deterioration. The survival curves estimated with KM method after the mid‐point imputation are shown in Figure 6. The two survival curves intersect around 15 months, corresponding to the early crossing survival curves scenario discussed in the previous section.

The p‐values for each test compared in the previous section are as follows: 0.0091 (RMST), 0.0010 (WMST ($\tau_{0}=15$)), 0.0011 (log‐rank), and 0.0001 (FH). As in the previous section, WMST is tested by shifting $\tau_{0}$ around the intersection point of the survival curves ($\tau_{0}\in \left\{12.5,17.5\right\}$). The resulting p‐values are 0.0021 (WMST ($\tau_{0}=12.5$)) and 0.0004 (WMST ($\tau_{0}=17.5$)). As a result, the p‐values for all tests are below 0.05, indicating a significant difference in survival rates between the radiation‐only group and the combination therapy group. It is well known that under the NPH, the log‐rank test and FH test cannot provide the clinically meaningful interpretation of the difference between the survival curves of the two groups. In contrast, for RMST, the difference between the groups is estimated to be 7.06 (95% CI [1.76,12.37]), and for WMST, the difference between the groups is estimated to be 7.53 (95% CI [3.06,12.00]). Furthermore, in this case, as the survival curves crossed early, the p‐value for WMST is lower than that for RMST.

## 5 Conclusion

In recent years, the NPH survival scenarios have been steadily increasing in cancer clinical trials. In particular, cancer immunotherapies, which have gained significant attention, often exhibit late differences and early crossing survival curves scenarios. Furthermore, in phase III trials targeting advanced cancers, the PFS is increasingly being adopted as a co‐primary endpoint alongside the OS. In response to these trends, we propose a class of methods for the estimation and testing of WMST designed for interval‐censored data and conduct extensive numerical simulations to compare their performance with conventional methods.

Through numerical simulations, WMST estimation method that uses mid‐point imputation followed by KM estimation (mid‐point + KM) demonstrates estimation accuracy comparable to that of WMST estimation method with Turnbull, which is the NPMLE. Unlike the frequently used right‐point + KM method in clinical practice, WMST estimation methods based on the mid‐point + KM and Turnbull are shown to be robust to both the proportion of interval‐censored cases and the interval widths of the interval‐censored data. Considering these findings, we establish WMST estimation based on mid‐point + KM as the standard approach. While achieving comparable statistical accuracy to Turnbull's method, this approach offers decisive practical advantages: it provides closed‐form variance estimation through Greenwood's formula, enables direct use of existing WMST software packages, and avoids the computational burden of bootstrap procedures. These characteristics make interval‐censored WMST analysis accessible for routine clinical research.

Numerical simulations for hypothesis testing show that even in the presence of interval‐censored data, WMST maintains higher power than FH (0,1) under the PH survival scenarios. More importantly, our comprehensive comparisons with the MaxCombo test—which represents current best practice for handling non‐proportional hazards in immunotherapy trials—reveal that while MaxCombo may achieve higher power in certain scenarios, WMST offers competitive performance with the distinct advantage of providing clinically interpretable effect estimates. Furthermore, WMST outperforms RMST in terms of power under late difference and early crossing survival curves scenarios, which are frequently observed in cancer immunotherapy trials. In the late difference and early crossing survival curves scenarios, the impact of selecting $\tau_{0}$ is investigated. It is found that when $\tau_{0}$ is appropriately selected relative to the divergence or crossing point of the two curves, WMST demonstrates improved power that, in certain scenarios, approaches that of MaxCombo, with the crucial benefit of maintaining clinical interpretability through its expected survival time estimate. Even when $\tau_{0}$ is set earlier than the optimal point, WMST maintains higher power than RMST for interval‐censored data. Our findings highlight that the proposed method represents a valuable addition to the methodological toolkit for assessing PFS in cancer immunotherapy trials, particularly under late difference and early crossing survival curves scenarios, by successfully balancing statistical efficiency with clinical interpretability.

An important direction for future research would be the development of versatile WMST methods specifically designed for interval‐censored data. As demonstrated by Paukner and Chappell [47] for right‐censored data, versatile testing approaches that combine multiple window specifications can substantially improve robustness against window misspecification. However, extending these approaches to interval‐censored data presents significant methodological and computational challenges, requiring comprehensive evaluation across numerous window combinations and censoring patterns. This represents a substantial research undertaking that warrants dedicated investigation in future work.

In this study, we employ the mid‐point imputation for handling interval‐censored data. However, the mid‐point imputation assigns a single value to all subjects whose disease progression (DP) is observed within the same interval. This means that all DP events within the same interval occur on the same day, which is unrealistic. To address this issue, Nakagawa and Sozu [53] propose an enhanced mid‐point imputation, and their numerical simulations demonstrate that the enhanced method provides more accurate estimates of the survival function compared to the standard mid‐point imputation. While applying the enhanced method in our study could lead to better WMST estimation accuracy and statistical power, a rigorous evaluation of these potential improvements remains an open question for future research.

## Author Contributions

All authors contributed equally to all aspects of this research, including conceptualization, methodology development, formal analysis, writing, and revision of the manuscript. T.M. served as the corresponding author and handled project administration. S.A. provided supervision. All authors have read and approved the final manuscript.

## Funding

The authors have nothing to report.

## Conflicts of Interest

The authors declare no conflicts of interest.

## Supporting information


**Data S1:** Supporting Information.

## Data Availability

Data sharing is not applicable as no new data were created in this study. The breast cosmesis (BCOS) dataset analyzed in Section 4 is publicly available in the R package interval. The R code implementing the window mean survival time methodology for interval‐censored data, as demonstrated in Section 4, is provided as Supporting Information accompanying this article.

## Appendix A Additional Simulation Scenarios

These supplementary scenarios extend the validation beyond Section 3.3's representative cases, ensuring robustness across diverse baseline hazard patterns. Additional null scenarios include Weibull distributions with decreasing (A‐i: p=0.5) and increasing (A‐ii: p=2) hazards. Scenario (A‐iii) features time‐varying piecewise‐linear hazards transitioning from constant to increasing rates. Supplementary alternative hypothesis scenarios maintain proportional hazards with varying baseline patterns: Weibull with decreasing (A‐iv) and increasing (A‐v) hazards, and piecewise‐linear with time‐varying rates (A‐vi), testing WMST's consistency across different hazard shapes.

The distributions or hazard functions of survival times under each scenario for both the null and alternative hypotheses are as follows.

- Null hypotheses
  – Weibull
    ∘ (A‐i) $T_{0},T_{1}∼\text{Weibull}\left(\lambda =1,p=0.5\right)$
    ∘ (A‐ii) $T_{0},T_{1}∼\text{Weibull}\left(\lambda =1,p=2\right)$
  – Piecewise‐linear hazard
    ∘ (A‐iii) $h_{0}\left(t\right)=h_{1}\left(t\right)=1$ (t∈[0,0.5)); $h_{0}\left(t\right)=h_{1}\left(t\right)=2t$ (t∈[0.5,1])
- Alternative hypotheses
  – Weibull
    ∘ (A‐iv) $T_{0}∼\text{Weibull}\left(\lambda =0.5,p=0.5\right),\;T_{1}∼\text{Weibull}\left(\lambda =1,p=0.5\right)$
    ∘ (A‐v) $T_{0}∼\text{Weibull}\left(\lambda =0.75,p=2\right),\;T_{1}∼\text{Weibull}\left(\lambda =1,p=2\right)$
  – Piecewise‐linear hazard
    ∘ (A‐v) $h_{0}\left(t\right)=1.5,h_{1}\left(t\right)=1$ (t∈[0,0.5)); $h_{0}\left(t\right)=3t,\;h_{1}\left(t\right)=2t$ (t∈[0.5,1])

**TABLE A1:** Size and power comparison of WMST tests with existing methods (RMST, Log‐rank, FH, and MaxCombo) across survival scenarios.

	RMST			WMST ($\tau_{0}=0.25$)			WMST ($\tau_{0}=0.50$)					
	Diff			Diff			Diff					
Distribution	True	Est.		True	Est.		True	Est.		Log‐rank	FH^a^	MaxCombo
Under $H_{0}$			Size			Size			Size	Size	Size	Size
Weibull (A‐i)	0.0000	0.0002	0.0514	0.0000	0.0002	0.0516	0.0000	0.0002	0.0535	0.0518	0.0512	0.0509
Weibull (A‐ii)	0.0000	0.0000	0.0530	0.0000	0.0000	0.0523	0.0000	0.0001	0.0517	0.0504	0.0495	0.0478
Piecewise‐linear hazard (A‐iii)	0.0000	0.0003	0.0542	0.0000	0.0002	0.0541	0.0000	0.0002	0.0535	0.0532	0.0546	0.0540
Under $H_{1}$			Power			Power			Power	Power	Power	Power
Weibull (A‐iv)	0.1154	0.1103	0.5086	0.0933	0.0913	0.5085	0.0631	0.0613	0.4983	0.5091	0.4791	0.4844
Weibull (A‐v)	0.1216	0.1174	0.8470	0.1178	0.1137	0.8577	0.0952	0.0911	0.8749	0.8753	0.8477	0.8553
Piecewise‐linear hazard (A‐vi)	0.1133	0.1081	0.6242	0.1006	0.0969	0.6415	0.0716	0.0684	0.6489	0.6510	0.6040	0.6301

*Note:* H0: (A‐i)–(A‐iii), H1: (A‐iv)–(A‐vi). This simulation is conducted at a significance level of α=0.05, with n=100 per arm, based on 10 000 replications.

^a^ FH is the Fleming‐Harington test.

Table A1 summarizes the results for these supplementary scenarios, confirming that WMST maintains appropriate Type I error control (scenarios A‐i–A‐iii) and competitive power (scenarios A‐iv–A‐vi) across varying baseline hazard patterns, supporting the robustness of our main conclusions.

## Appendix B Complete Simulation Results

Tables B1, B2, B3 contain comprehensive numerical results corresponding to Figures 3, 4, 5 in the main text, providing detailed values for reproducibility and precise comparisons.

**TABLE B1:** Size and power comparison of WMST tests with existing methods (RMST, Log‐rank, FH, and MaxCombo) across 11 survival scenarios.

	RMST			WMST ($\tau_{0}=0.25$)			WMST ($\tau_{0}=0.50$)					
	Diff			Diff			Diff					
Distribution	True	Est.		True	Est.		True	Est.		Log‐rank	FH^a^	MaxCombo
Under $H_{0}$			Size			Size			Size	Size	Size	Size
Weibull (i)	0.0000	0.0000	0.0540	0.0000	0.0001	0.0541	0.0000	0.0001	0.0540	0.0521	0.0521	0.0517
Piecewise‐linear hazard (ii)	0.0000	0.0000	0.0514	0.0000	0.0000	0.0502	0.0000	−0.0001	0.0509	0.0497	0.0509	0.0507
Under $H_{1}$ (PH)			Power			Power			Power	Power	Power	Power
Weibull (iii)	0.1998	0.1902	0.9753	0.1753	0.1686	0.9775	0.1224	0.1166	0.9756	0.9786	0.9664	0.9742
Piecewise‐linear hazard (iv)	0.1234	0.1159	0.7147	0.1025	0.0976	0.7064	0.0663	0.0622	0.6686	0.7130	0.6328	0.6871
Under $H_{1}$ (NPH)			Power			Power			Power	Power	Power	Power
Early difference (v)	0.1469	0.1387	0.8720	0.1143	0.1103	0.7840	0.0612	0.0590	0.5583	0.7236	0.3626	0.8381
Late difference (vi)	0.0974	0.0860	0.5058	0.0973	0.0857	0.6440	0.0861	0.0737	0.8823	0.7105	0.9627	0.9155
Crossing hazards (vii)	0.1397	0.1324	0.7710	0.1076	0.1044	0.6700	0.0538	0.0524	0.4005	0.5356	0.1755	0.7126
Crossing hazards (viii)	0.1259	0.1198	0.6721	0.1017	0.0984	0.5957	0.0519	0.0506	0.3482	0.4129	0.1521	0.5868
Early crossing survival curves (ix)	0.1550	0.1441	0.8743	0.1644	0.1508	0.9732	0.1248	0.1134	0.9929	0.9496	0.9941	0.9964
Middle crossing survival curves (x)	−0.0211	−0.0195	0.0791	0.0034	0.0010	0.0499	0.0210	0.0182	0.1209	0.0508	0.1743	0.2835
Late crossing survival curves (xi)	−0.0964	−0.0906	0.5306	−0.0601	−0.0592	0.3425	0.0140	−0.0148	0.0922	0.3160	0.0615	0.6663

*Note:* $H_{0}$: (i) and (ii), $H_{1}$ (PH): (iii) and (iv), $H_{1}$ (NPH): (v)–(xi). This simulation is conducted at a significance level of α=0.05, with n=100 per arm, based on 10 000 replications.

^a^ FH is the Fleming‐Harington test.

**TABLE B2:** Power for WMST tests based on $\tau_{0}$, the divergence point (x) of survival curves and difference of RMST under the late difference scenario, compared to three existing methods: RMST, log‐rank, FH, and MaxCombo.

		WMST												
	RMST	$\tau_{0}=0.05$	0.10	0.15	0.20	0.25	0.30	0.35	0.40	0.45	0.50	Log‐rank	FH^a^	MaxCombo
x=0.10														
*δ* = 0.10	0.5398	0.5426	0.5566	0.5922	0.6274	0.6764	0.7228	0.7782	0.8200	0.8568	0.8876	0.7404	0.9562	0.9154
*δ* = 0.15	0.8160	0.8190	0.8358	0.8590	0.8894	0.9230	0.9494	0.9688	0.9824	0.9904	0.9930	0.9474	0.9990	0.9972
*δ* = 0.20	0.9462	0.9476	0.9564	0.9678	0.9788	0.9882	0.9938	0.9970	0.9986	0.9992	0.9996	0.9930	1.0000	1.0000
x=0.15														
*δ* = 0.10	0.5458	0.5474	0.5654	0.5974	0.6364	0.6832	0.7338	0.7818	0.8224	0.8570	0.8924	0.7422	0.9558	0.9172
*δ* = 0.15	0.8104	0.8142	0.8298	0.8550	0.8860	0.9192	0.9498	0.9670	0.9810	0.9884	0.9924	0.9440	0.9992	0.9972
*δ* = 0.20	0.9552	0.9566	0.9636	0.9734	0.9822	0.9912	0.9950	0.9980	0.9988	0.9996	0.9996	0.9954	1.0000	1.0000
x=0.20														
*δ* = 0.10	0.5464	0.5504	0.5710	0.5992	0.6408	0.6858	0.7362	0.7872	0.8270	0.8616	0.8970	0.7448	0.9588	0.9204
*δ* = 0.15	0.8270	0.8306	0.8458	0.8728	0.8998	0.9302	0.9556	0.9734	0.9858	0.9908	0.9940	0.9522	0.9992	0.9976
*δ* = 0.20	0.9604	0.9628	0.9690	0.9768	0.9848	0.9924	0.9956	0.9984	0.9994	0.9996	1.0000	0.9958	1.0000	1.0000
x=0.25														
*δ* = 0.10	0.5436	0.5462	0.5674	0.5952	0.6370	0.6840	0.7362	0.7852	0.8258	0.8680	0.9024	0.7456	0.9638	0.9284
*δ* = 0.15	0.8308	0.8338	0.8470	0.8756	0.9014	0.9340	0.9578	0.9752	0.9864	0.9912	0.9954	0.9544	0.9992	0.9978
*δ* = 0.20	0.9646	0.9654	0.9692	0.9784	0.9862	0.9924	0.9962	0.9986	0.9994	0.9996	1.0000	0.9958	1.0000	1.0000
x=0.30														
*δ* = 0.10	0.5438	0.5466	0.5646	0.5940	0.6342	0.6852	0.7360	0.7892	0.8344	0.8796	0.9108	0.7550	0.9692	0.9388
*δ* = 0.15	0.8302	0.8324	0.8488	0.8734	0.9004	0.9314	0.9592	0.9768	0.9884	0.9936	0.9962	0.9564	0.9994	0.9982
*δ* = 0.20	0.9648	0.9662	0.9712	0.9800	0.9894	0.9934	0.9968	0.9986	0.9994	1.0000	1.0000	0.9972	1.0000	1.0000

*Note:* This simulation is conducted at a significance level of α=0.05, with n=100 per arm, based on 5000 replications.

^a^ FH is the Fleming‐Harington test.

**TABLE B3:** Power for WMST tests based on $\tau_{0}$, the crossing point (x) of survival curves and difference of RMST under the early crossing survival curves scenario, compared to three existing methods: RMST, log‐rank, FH, and MaxCombo.

		WMST												
	RMST	$\tau_{0}=0.05$	0.10	0.15	0.20	0.25	0.30	0.35	0.40	0.45	0.50	Log‐rank	FH^a^	MaxCombo
x=0.10														
*δ* = 0.10	0.6012	0.6074	0.6320	0.6554	0.6794	0.6912	0.7046	0.7114	0.7134	0.7112	0.7086	0.6422	0.6692	0.7114
*δ* = 0.15	0.8634	0.8688	0.8866	0.9064	0.9242	0.9356	0.9428	0.9486	0.9500	0.9514	0.9504	0.9032	0.9350	0.9536
*δ* = 0.20	0.9756	0.9762	0.9818	0.9858	0.9914	0.9930	0.9952	0.9960	0.9960	0.9962	0.9970	0.9858	0.9958	0.9976
x=0.15														
*δ* = 0.10	0.5834	0.5950	0.6372	0.6772	0.7124	0.7378	0.7600	0.7742	0.7856	0.7888	0.7930	0.6586	0.7624	0.8214
*δ* = 0.15	0.8678	0.8732	0.8956	0.9202	0.9400	0.9550	0.9646	0.9690	0.9716	0.9744	0.9756	0.9190	0.9698	0.9832
*δ* = 0.20	0.9764	0.9784	0.9846	0.9898	0.9934	0.9964	0.9972	0.9974	0.9976	0.9984	0.9984	0.9902	0.9992	0.9992
x=0.20														
*δ* = 0.10	0.5670	0.5776	0.6264	0.6848	0.7362	0.7756	0.8094	0.8348	0.8482	0.8546	0.8624	0.6946	0.8528	0.8990
*δ* = 0.15	0.8614	0.8674	0.8936	0.9248	0.9504	0.9676	0.9784	0.9838	0.9860	0.9892	0.9906	0.9358	0.9900	0.9952
*δ* = 0.20	0.9766	0.9772	0.9834	0.9918	0.9958	0.9978	0.9984	0.9984	0.9990	0.9994	0.9998	0.9942	0.9998	1.0000
x=0.25														
*δ* = 0.10	0.7726	0.7818	0.8272	0.8750	0.9220	0.9508	0.9682	0.9758	0.9802	0.9816	0.9828	0.9084	0.9780	0.9938
*δ* = 0.15	0.8504	0.8582	0.8926	0.9298	0.9586	0.9770	0.9864	0.9914	0.9946	0.9962	0.9972	0.9572	0.9974	0.9992
*δ* = 0.20	0.9728	0.9746	0.9824	0.9918	0.9966	0.9984	0.9992	0.9996	0.9998	1.0000	1.0000	0.9966	1.0000	1.0000
x=0.30														
*δ* = 0.10	0.5542	0.5694	0.6360	0.7238	0.8062	0.8724	0.9218	0.9492	0.9654	0.9758	0.9800	0.8318	0.9844	0.9970
*δ* = 0.15	0.8472	0.8552	0.8948	0.9376	0.9680	0.9852	0.9928	0.9974	0.9980	0.9988	0.9990	0.9788	0.9998	1.0000
*δ* = 0.20	0.9688	0.9710	0.9806	0.9920	0.9966	0.9990	0.9998	1.0000	1.0000	1.0000	1.0000	0.9982	1.0000	1.0000

*Note:* This simulation was conducted at a significance level of α=0.05, with n=100 per arm, based on 5000 replications.

^a^ FH is the Fleming‐Harington test.
